# Neuroprotective and Anti-Inflammatory Effect of Pterostilbene Against Cerebral Ischemia/Reperfusion Injury *via* Suppression of COX-2

**DOI:** 10.3389/fphar.2021.770329

**Published:** 2021-11-02

**Authors:** Wenjun Yan, Dongqing Ren, Xiaoxue Feng, Jinwen Huang, Dabin Wang, Ting Li, Dong Zhang

**Affiliations:** Department of Anesthesiology, Gansu Provincial Hospital, Lanzhou, China

**Keywords:** cerebral ischemia reperfusion, pterostilbene, inflammation, oxidative stress, COX-2

## Abstract

**Background:** The incidence of cerebral ischemia disease leading cause of death in human population worldwide. Treatment of cerebral ischemia remains a clinical challenge for researchers and mechanisms of cerebral ischemia remain unknown. During the cerebral ischemia, inflammatory reaction and oxidative stress plays an important role. The current investigation scrutinized the neuroprotective and anti-inflammatory role of pterostilbene against cerebral ischemia in middle cerebral artery occlusion (MCAO) rodent model and explore the underlying mechanism.

**Methods:** The rats were divided into following groups viz., normal, sham, MCAO and MCAO + pterostilbene (25 mg/kg) group, respectively. The groups received the oral administration of pterostilbene for 30 days followed by MCAO induction. The neurological score, brain water content, infarct volume and Evan blue leakage were estimated. Hepatic, renal, heart, inflammatory cytokines and inflammatory mediators were estimated.

**Results:** Pterostilbene treatment significantly (*p < 0.001*) improved the body weight and suppressed the glucose level and brain weight. Pterostilbene significantly (*p < 0.001*) reduced the hepatic, renal and heart parameters. Pterostilbene significantly (*p* < 0.001) decreased the level of glutathione (GSH), glutathione peroxidase (GPx), superoxide dismutase (SOD) and decreased the level of malonaldehyde (MDA), 8-Hydroxy-2′-deoxyguanosine (8-OHdG). Pterostilbene significantly (*p* < 0.001) inflammatory cytokines and inflammatory parameters such as cyclooxygenase-2 (COX-2), inducible nitric oxidase synthase (iNOS) and prostaglandin (PGE_2_). Pterostilbene significantly (*p* < 0.001) down-regulated the level of metalloproteinases (MMP) such as MMP-2 and MMP-9. Pterostilbene suppressed the cellular swelling, cellular disintegration, macrophage infiltration, monocyte infiltration and polymorphonuclear leucocyte degranulation in the brain.

**Conclusion:** In conclusion, Pterostilbene exhibited the neuroprotective effect against cerebral ischemia in rats *via* anti-inflammatory mechanism.

## Introduction

Stroke is a complicated disease that causes the number of death and disability whole over the world, and it is accompanied by cognitive dysfunctions (Vakili et al., 2014; Wang K. et al., 2020). According to the World Health Organization (WHO), over 15 million people are affected by stroke (Alva-Díaz et al., 2020). Ischemia stroke is the most frequent type of stroke, accounting for 87 percent of all instances. It is caused by thromboembolic blockage of cerebral arteries, which causes an ischemic cascade and tissue injury (Özön and Cüce, 2020; Tuo et al., 2021). Cerebral ischemia disease is the major neurological disease worldwide and incidence of cerebral ischemia rapidly increases last few decades (Yuan et al., 2020). The treatment of cerebral ischemia still challenge to the researcher (Wang et al., 2018; Leung et al., 2020). But few research suggest that neurovascular units involve in the disease (Pan et al., 2018) and the researcher targeting the neurovascular unit (NVU) for the treatment of overall disease, which exhibited the protective against the neuronal damage, neurons and blood brain barrier (BBB) such as extracellular matrix, vascular endothelial cells, microglia and astrocytes (Crofts et al., 2020; Lake et al., 2017).

During the ischemic cascade, oxygen and energy deprivation start the production of reactive oxygen species (ROS), followed through inflammatory reaction, deposition of intracellular calcium and glutamate excitotoxicity (Michalski et al., 2017; Tuo et al., 2021). Ischemic tissue reperfusion enhances the neuroinflammatory reaction and production of ROS. Various studies have shown that oxidative stress is linked to neuronal cell death in ischemic lesions (Patel et al., 2020; Yan et al., 2020). ROS such as nitric oxide (NO), hydrogen peroxide (H_2_O_2_), superoxide anion (O_2_) and hydroxyl free radicals destroys the cellular structures includes nucleic acid, proteins, lipids, redox sensitive enzymes, membrane receptors and channels, which eventually induces the neuronal injury in the ischemic lesions (Jin et al., 2016; Liang et al., 2019; Leung et al., 2020). Furthermore, the increased proportion of ROS leakage from the mitochondrial cytochrome C, which further recruits caspases, worsening neuronal death after ischemia and reperfusion injury (Tang et al., 2016; Han et al., 2018; Zhang et al., 2020). Due to increase the level of ROS leading the cell death, inflammatory reaction and neural dysfunction after the reperfusion (Zhang et al., 2018; Leung et al., 2020). Following cerebral ischemia, an inflammatory response plays a key role in the development of secondary brain injury. Inflammatory mediators include COX-2, nuclear factor kappa B (NF-κB) and MMPs play a significant role in the expansion of cerebral ischemic (Li et al., 2017; Zhang et al., 2018). During the cerebral ischemia injury, the level of above discuss enzymes boosted to considerable level in the brain area. In the treatment of cerebral ischemia, antioxidants and anti-inflammatory agents are helpful (Liang et al., 2020).

Extracellular Matrix (ECM) degrading enzymes such as MMP-2 and MMP-9, are most directly linked to BBB degradation (Traystman, 2003; Ma et al., 2020). The inhibition of ECM degrading enzymes considerably suppresses the cerebral infarct volume and cerebral edema induced *via* ischemia and suppress the BBB injury (Turner and Sharp, 2016). Previous study showed that the reperfusion is the protective in suppressing the ischemic brain injury and also showed the beneficial effect for recovering the various reversible injury (Gresoiu and Christou, 2020; Yu et al., 2020). Though, few research suggest that the blood reperfusion for ischemic tissue could causes the dysfunction and injury in some cases (Gresoiu and Christou, 2020). Furthermore, consideration is always taken of how to inhibit reperfusion injury during the treatment of ischemic stroke (Vakili et al., 2014). During the ischemic reperfusion stop or abrupt the blood supply in the brain area which resultant shortage the oxygen and glucose level in the brain that leading to the anaerobic pathway of glycolytic cycle. The glycolytic cycle generate the high H+ ion, lactic acid and reversal to the mitochondrial matrix (Liang et al., 2019; Wang et al., 2020a; Zhao et al., 2020). All the cascade suppressed the cytoplasmic pH and increase the formation of free radicals. Due to continuous production of free radicals, its finally induces the oxidative stress and start the inflammatory reaction (Li et al., 2017; Zhang et al., 2018).

More than hundred traditional Chinese medicine (TCM) patents have been filed in China in the last few years for the treatment of ischemic stroke, including therapies for ischemia reperfusion injury (Gupta et al., 2010; Peng et al., 2019; Singh et al., 2020). Dietary phytochemicals getting more popularity due to its wide range of benefits includes regulation of immune system, anti-oxidation and suppression of inflammation. Resveratrol (polyphenol flavonoids) isolated from the skin of red grape and suggested the protective effect against various inflammatory reaction. Pterostilbene (*trans*-3,5-dimethoxy-4′-hydroxystilbene) is a stilbene, that is naturally occurring methoxylated analog of Revesterol, commonly found in blueberry (Acharya and Ghaskadbi, 2013; Shi et al., 2019). Pterostilbene shows the higher bioactivity and bioavailability than resveratrol due to presence of two extra methyl group in the structure (Supplementary Figure S1). The structure of pterostilbene showed the removal of two hydroxy group with two methoxy group, which increase the bioactivity and oral bioavailability and demonstrate the prolong metabolism (Wu et al., 2017; Yang et al., 2017; Shi et al., 2019). Pterostilbene having the bioavailability (85%) as compared to the bioavailability of revesterol (20%) in animal model. Additionally, clinical studies showed that the pterostilbene may be a potent anti-inflammatory drug against various diseases (Acharya and Ghaskadbi, 2013; Shi et al., 2019; Zhang et al., 2021). In this experimental study, we try to explore the neuroprotective and anti-inflammatory effect of pterostilbene against the MCAO rat model.

## Material and Methods

### Experimental Rodent

Swiss Wistar rats (weight 200 ± 20 g, sex-both, aged 8–10 weeks) were used in this study. The whole experimental study was carried out using the institutional animal ethical committee guidelines. All the rats were kept in the standard laboratory condition 20 ± 5°C temperature, 12 h dark and 12 h light cycle and 65% relative humidity. All the rats were received the standard pellet (rat chow) and water *ab libitum*. The rats were kept 7 days for acclimatization for adopting the laboratory condition.

### MCAO/R Method

Briefly, the rats were anesthetized using the 50 mg/kg intraperitoneal injection of ketamine hydrochloride and 5 mg/kg xylazine and monitored accordingly. The common carotid artery was isolated and the branches of external carotid artery (right) were carefully removed (Pan et al., 2018). To obstruct the source of MCAO, a nylon monofilament suture (4–0) with a silicon coated tip was pushed to the internal carotid artery (Yuan et al., 2020). The same operation was performed on the normal group (sham group), but no suture was put. After cerebral ischemia (2 h), the suture was removed for reperfusion (24 h). The rats were divided into different groups such as Group I: control; Group II: Sham; Group III: MCAO and Group IV: MCAO + pterostilbene (35 mg/kg), respectively.

### Neurological Deficits

Neurological dysfunction were assessed after 2 h of MCAO and then every day for the next 5 days (Shamim and Khan, 2019). The behavioural impairments and post chemical motor were examined using a 4-point neuro score (Yuan et al., 2020). The grade for neurological impairments was as follows:

Symptoms are absent in grade 0.

Grade 1: bending of the forelimbs
Grade 2: reduced lateral push (and forelimb flexion) resistance without circling
Grade 3: circle and the same conduct as grade 2.

### Blood and Tissue Sampling

The rats were euthanized at the end of the protocol so that blood (plasma/serum) and organs (liver and brain) could be collected and kept at 80°C. All the blood and tissue samples kept for the histopathological evaluation and biochemical estimation.

### BBB Permeability

For the determination of BBB permeability, the all-group rats were received the Evans blue (2%) in the tail vein before the sacrifice (2 h). after that removed the brain tissue and weighted and placed into the 1 ml dimethylformamide and incubated for next 24 h at 60°C. Afterthat centrifuge at 1 g rpm for 10 min to separate the supernatant (Pan et al., 2018). The supernatant collected and take absorbance at 620 nm wavelength using the spectrophotometer.

### Cerebral Edema

For the determination of cerebral edema, the brain water content was estimated using the dry weight method of previous reported method with minor modification (Yang et al., 2020). Briefly, the brain tissue was successfully removed after the reperfusion (24 h) and weight immediately and baked in the oven for 24 h at 120°C and again weight.

The brain water content was estimated using the following formula

$$Brain\;water\;content=\frac{\left(a-b\right)}{a}\;X\;100.$$

### Biochemical Assays

Turbid Metric Immunoassay model was used for the estimation of serum C-reactive protein (CRP) using the kit (Conformidad Europea, Spain).

Enzymatic colorimetric GOD-POD kit was used for the estimation of blood glucose (Global, United Kingdom). Lactate dehydrogenase (LDH) assay kit was used for the determination of LDH activity (Institute of Biological Engineering of Nanjing Jiancheng, Nanjing, China) following the manufacture protocol.

For the estimation of lipid parameters such as high-density lipoprotein cholesterol (HDL-c), triglyceride (TG) and cholesterol (TC) was estimated using the enzymatic kit kit (Institute of Biological Engineering of Nanjing Jiancheng, Nanjing, China). The low-density lipoprotein cholesterol (LDL-c) and very low-density lipoprotein cholesterol (VLDL-c) were determined using the Friedewald’s formula (Mendes de Cordova et al., 2018).

Berthelot colorimetric model was used for the estimation of urea concentration in the serum. Jaffe’s method was used for the determination of creatinine using the enzymatic kit (Biogene Diagnostics, United States). Blood urea nitrogen (BUN) and uric acid was estimated using the previous reported method with minor modification (Pandey et al., 2018; Kumar et al., 2021).

The hepatic parameters include alkaline phosphatase (ALP), alanine aminotransferase (ALT) and aspartate aminotransferase (AST) using the enzymatic kit (Institute of Biological Engineering of Nanjing Jiancheng, Nanjing, China).

### Antioxidant Parameters

The antioxidant parameters include SOD, GSH, GPx, MDA and CAT were estimated using the previous reported method with minor modification (Bhatt et al., 2017, 2018).

### MMP Levels

MMP-2 and MMP-9 were estimated using the gelatinase assay kit (Chemicon International, Inc. Temecula, CA, United States) following the manufacture protocol.

### Inflammatory Cytokines

Pro-inflammatory cytokines such as interleukin-1β (IL-1β), interleukin-4 (IL-4), tumor necrosis factor-α (TNF-α), interleukin-6 (IL-6), interleukin-1 (IL-10) and interleukin-1 (IL-1) were estimated using the Enzyme-Linked Immunosorbent Assay Kits (ELISA) (Institute of Biological Engineering of Nanjing Jiancheng, Nanjing, China) following the manufacture protocol.

### Inflammatory Mediators

Inflammatory mediators such as NF-κB, i-NOS, NO, COX-2 and PGE2 using the Enzyme-Linked Immunosorbent Assay Kits (ELISA) (Institute of Biological Engineering of Nanjing Jiancheng, Nanjing, China) following the manufacture protocol.

### Statistical Analysis

The current study’s findings were given as a mean standard error mean (S.E.M.). The statistical analysis was performed using Graphpad Prism 7. The difference between the intergroups was determined using a post hoc *t* test. The significance level was set at *p < 0.05*.

## Results

### Effect of Pterostilbene on Neurological Parameter

**FIGURE 1:**
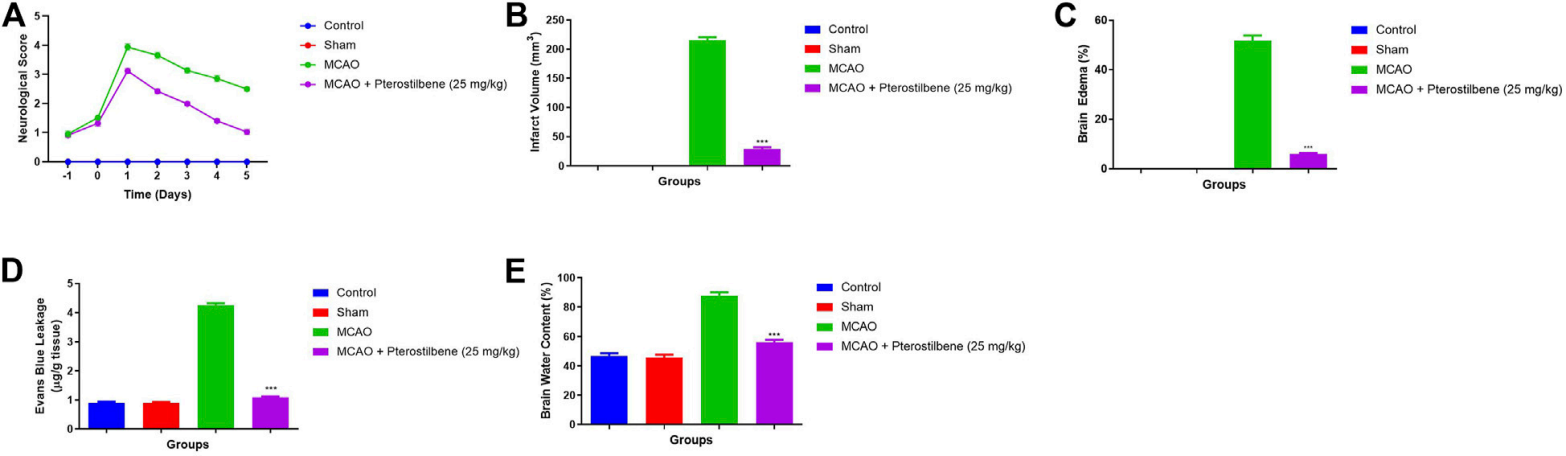
showed the neurological parameter and score of among experimental groups. **(A)** neurological score, **(B)** infarct volume, **(C)** brain edema, **(D)** evans blue leakage and **(E)** brain water content. Values are expressed as mean ± SEM. The *t*-test was used for the significant difference between groups: *p < 0.05, **p < 0.01, ***p < 0.005. NS, non-significant.

During cerebral ischemia, it increases the neurological score. A neurological score of 0 was observed in the normal and sham group rats. The MCAO group rats showed an enhanced neurological score, which started from day 0 and reached its maximum on day 1 and remained at the higher end of the protocol (day 5). Pterostilbene treated rats significantly (*p* < 0.001) suppressed the neurological score. Pterostilbene treated rats exhibited a reduced neurological score at different time intervals (day 0, 1, and 5) as compared to the MCAO group (Figure 1A).

Infarct volume (Figure 1B), brain edema (Figure 1C), Evans blue leakage (Figure 1D) and brain water content (Figure 1E) were higher observed in the MCAO group rats. Pterostilbene treated rats exhibited the suppressed level of infarct volume, brain edema, Evans blue leakage and brain water content.

### Effect of Pterostilbene on Body Weight, Glucose Level and Organ Weight

**FIGURE 2:**
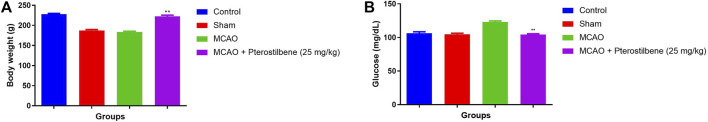
showed the body weight and glucose level of among experimental groups. **(A)** body weight and **(B)** blood glucose level. Values are expressed as mean ± SEM. The *t*-test was used for the significant difference between groups: *p < 0.05, **p < 0.01, ***p < 0.005. NS, non-significant.

The body weight was slightly reduced in the sham control and MCAO group rats. MCAO group rats receiving pterostilbene significantly (*p* < 0.001) improved their body weight (Figure 2A).

The blood glucose remained constant in the normal and sham control group rats. The blood glucose level slightly decreased in the MCAO group. Pterostilbene treated group rats improved the blood glucose level (Figure 2B).

**FIGURE 3:**
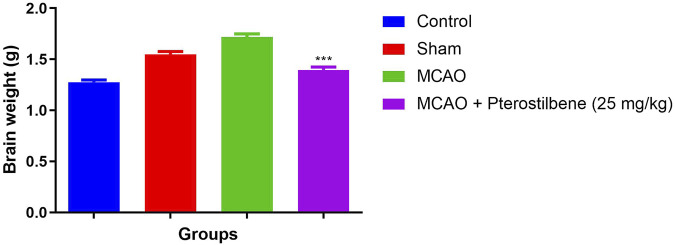
showed the brain weight of among experimental groups. Values are expressed as mean ± SEM. The *t*-test was used for the significant difference between groups: *p < 0.05, **p < 0.01, ***p < 0.005. NS, non-significant.

The brain weight slightly enhanced in the sham group rats as compared to the normal rats. MCAO group rats exhibited the increased brain weight as compared to all experimental group. MCAO group rats treated with the pterostilbene treated group rats demonstrated the reduced brain weight (Figure 3).

### Effect of Pterostilbene on LDH and CRP

**FIGURE 4:**
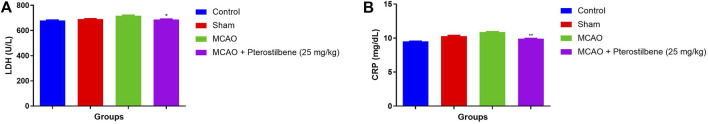
showed the LDH and CRP level of among experimental groups. **(A)** LDH and **(B)** CRP. Values are expressed as mean ± SEM. The *t*-test was used for the significant difference between groups: *p < 0.05, **p < 0.01, ***p < 0.005. NS, non-significant.

The level of LDH (Figure 4A) and CRP (Figure 4B) were estimated in the different groups. The level of LDH (691 ± 12.45 U/L) and CRP (10.89 ± 1.32 mg/dl) slightly enhanced in the MCAO group and pterostilbene treated rats significantly (*p < 0.05*) reduced the level of LDH (687 ± 11.34 U/L) and CRP (9.91 ± 1.93 mg/dl).

### Effect of Pterostilbene on Hepatic Parameter

**FIGURE 5:**
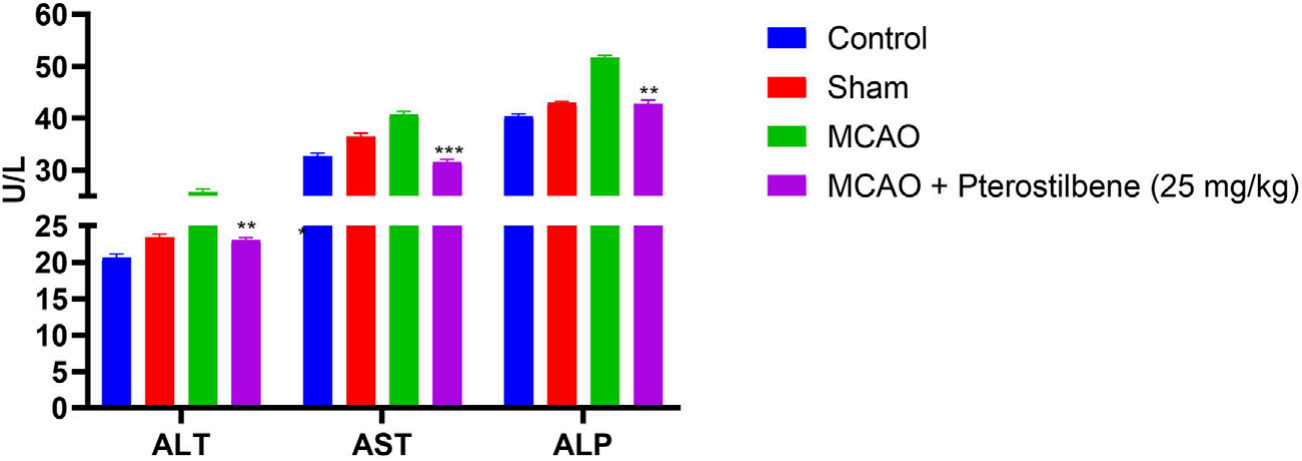
showed the hepatic parameters of among experimental groups. Values are expressed as mean ± SEM. The *t*-test was used for the significant difference between groups: *p < 0.05, **p < 0.01, ***p < 0.005. NS, non-significant.

MCAO group rats displayed the boosted level of ALT (25.45 ± 2.38 U/L), AST (40.73 ± 4.93 U/L) and ALP (51.61 ± 2.83 U/L) as compared to normal and sham group rats. After the pterostilbene treatment reduced the level of hepatic parameters ALT (23.04 ± 2.93 U/L), AST (31.59 ± 3.82 U/L) and ALP (42.82 ± 3.89 U/L) (Figure 5).

### Effect of Pterostilbene on Renal Parameters

**FIGURE 6:**
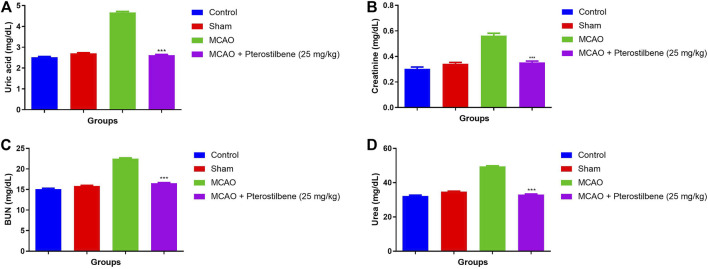
showed the renal parameters of among experimental groups. **(A)** uric acid, **(B)** creatinine, **(C)** BUN and **(D)** urea. Values are expressed as mean ± SEM. The *t*-test was used for the significant difference between groups: *p < 0.05, **p < 0.01, ***p < 0.005. NS, non-significant.

The levels of Uric acid (4.67 ± 0.83 mg/dl) (Figure 6A), creatinine (0.56 ± 0.03 mg/dl) (Figure 6B), BUN (22.51 ± 1.34 mg/dl) (Figure 6C) and urea (49.46 ± 3.94 mg/dl) (Figure 6D) were observed in different experimental group. MCAO group rats demonstrated the increased level of uric acid (2.62 ± 0.67 mg/dl), creatinine (0.35 ± 0.04 mg/dl), BUN (16.54 ± 2.32 mg/dl) and urea (33.05 ± 2.93 mg/dl), which was significantly (*p* < 0.001) diminished after the pterostilbene treatment.

### Effect of Pterostilbene on NO Level

**FIGURE 7:**
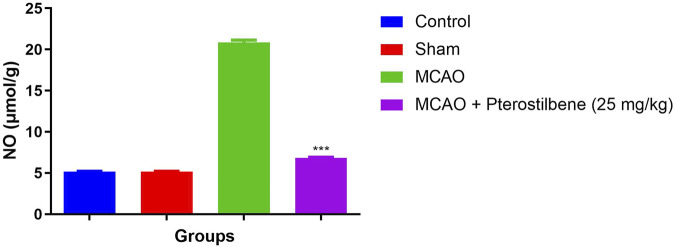
showed the level of nitric oxide of among experimental groups. Values are expressed as mean ± SEM. The *t*-test was used for the significant difference between groups: *p < 0.05, **p < 0.01, ***p < 0.005. NS, non-significant.

The NO level was considerably boosted in the MCAO group rats as compared to normal and sham control. MCAO group rats received the NO significantly (*p* < 0.001) repressed the level (Figure 7).

### Effect of Pterostilbene on Lipid Parameters

**FIGURE 8:**
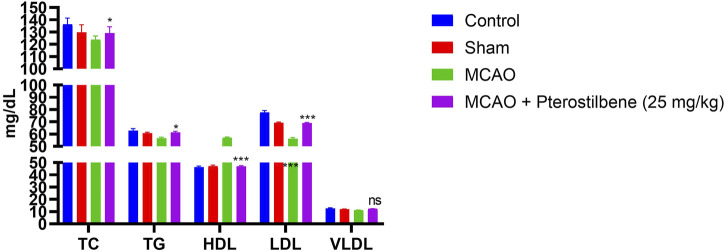
showed the lipid parameters of among experimental groups. Values are expressed as mean ± SEM. The *t*-test was used for the significant difference between groups: *p < 0.05, **p < 0.01, ***p < 0.005. NS, non-significant.

Figure 8 exhibited the lipid parameters in experimental groups. The level of lipid parameters such as TG, LDL, TC, VLDL boosted and HDL level reduced in the MCAO group. Pterostilbene treated rats exhibited the reduced level of TG, LDL, TC, VLDL and enhanced level of HDL.

### Effect of Pterostilbene on Antioxidant Parameters

**FIGURE 9:**
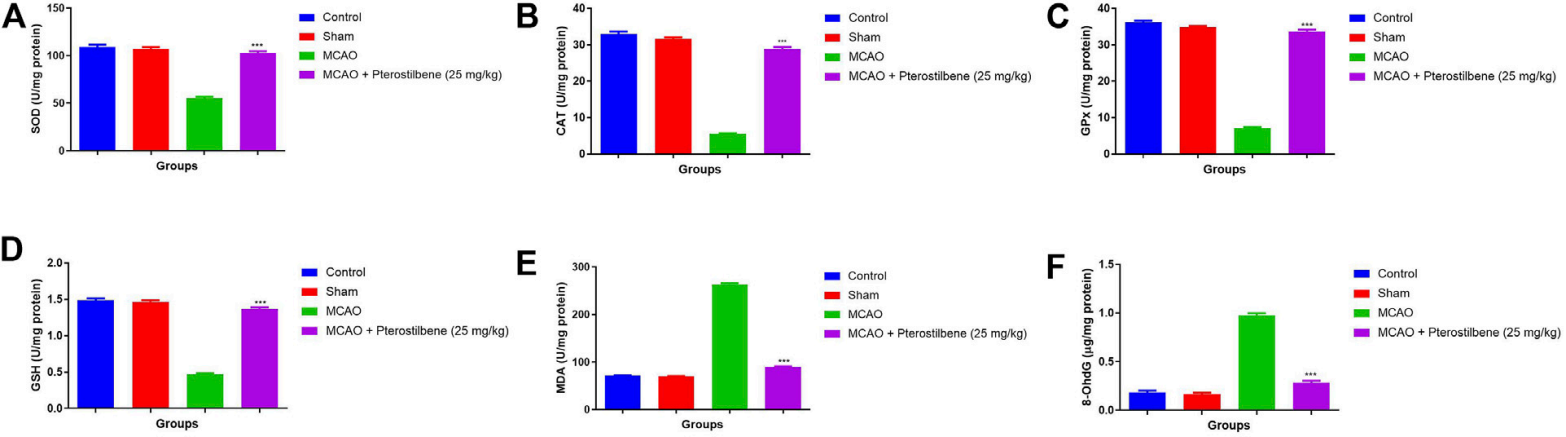
showed the antioxidant parameters of among experimental groups. **(A)** SOD, **(B)** CAT, **(C)** GPx, **(D)** GSH, **(E)** MDA and **(F)** 8-OhdG. Values are expressed as mean ± SEM. The *t*-test was used for the significant difference between groups: *p < 0.05, **p < 0.01, ***p < 0.005. NS, non-significant.

Figure 9 exhibited the antioxidant parameters of different group rats. MCAO group rats exhibited the decreased level of SOD (55.03 ± 5.32 U/mg protein) (Figure 9A), CAT (5.57 ± 0.83 U/mg protein) (Figure 9B), GPx (7.11 ± 0.93 U/mg protein) (Figure 9C), GSH (0.47 ± 0.04 U/mg protein) (Figure 9D) and enhanced level of MDA (263.4 ± 10.34 U/mg protein) (Figure 9E), 8OdhG OdhG (0.97 ± 0.09 μg/mg protein) (Figure 9F) as compared to normal and sham group rats. MCAO group rats treated with pterostilbene significantly (*p* < 0.001) improved the level of SOD (102.34 ± 7.54 U/mg protein), CAT (28.85 ± 1.33 U/mg protein), GPx (33.73 ± 4.34 U/mg protein), GSH (1.34 ± 0.45 U/mg protein) and suppressed the level of MDA (89.45 ± 3.45 U/mg protein), 8-OdhG (0.28 ± 0.05 μg/mg protein).

### Effect of Pterostilbene on Inflammatory Cytokines

**FIGURE 10:**
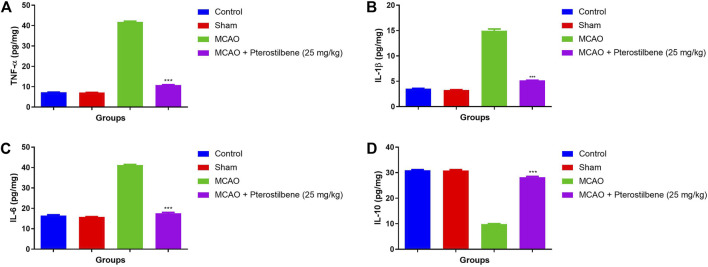
showed the inflammatory cytokines of among experimental groups. **(A)** TNF-α, **(B)** IL-1β, **(C)** IL-6 and **(D)** IL-10. Values are expressed as mean ± SEM. The *t*-test was used for the significant difference between groups: *p < 0.05, **p < 0.01, ***p < 0.005. NS, non-significant.

The level of inflammatory cytokines boosted during the cerebral ischemia due to expansion of inflammatory disease. MCAO group rats exhibited the increased level of TNF-α (41.34 ± 1.57 pg/mg) (Figure 10A), IL-1β (14.37 ± 1.23 pg/mg) (Figure 10B), IL-6 (41.45 ± 2.34 pg/mg) (Figure 10C) and reduced level of (9.89 ± 1.34 pg/mg) IL-10 (Figure 10D). MCAO group rats received the pterostilbene treatment significantly diminished the level of TNF-α (10.34 ± 0.89 pg/mg) (Figure 10A), IL-1β (5.23 ± 0.83 pg/mg) (Figure 10B), IL-6 (17.04 ± 2.93 pg/mg) (Figure 10C) and improved the level of IL-10 (28.34 ± 1.83 pg/mg) (Figure 10D).

### Effect of Pterostilbene on Inflammatory Mediators

**FIGURE 11:**
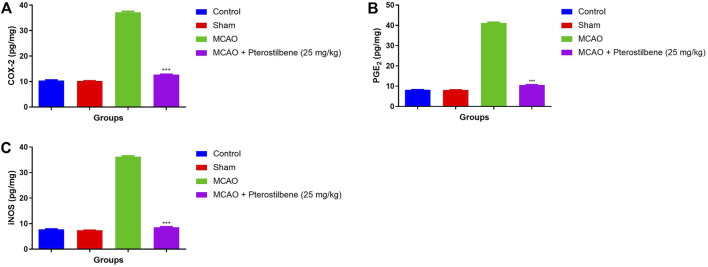
showed the inflammatory mediators of among experimental groups. **(A)** COX-2, **(B)** PGE_2_ and **(C)** iNOS. Values are expressed as mean ± SEM. The *t*-test was used for the significant difference between groups: *p < 0.05, **p < 0.01, ***p < 0.005. NS, non-significant.

Figures 11A–C showed the level of inflammatory parameters in different experimental group. MCAO group rats showed the improved level of COX-2 (37.34 ± 2.34 pg/mg) (Figure 11A), PGE_2_ (41.93 ± 3.23 pg/mg) (Figure 11B) and iNOS (36.54 ± 2.93 pg/mg) (Figure 11C) and pterostilbene treatment reduced the inflammatory parameters such as COX-2 (12.34 ± 0.45 pg/mg), PGE2 (10.02 ± 0.93 pg/mg) and iNOS (10.34 ± 1.83 pg/mg).

### Effect of Pterostilbene on MMP

**FIGURE 12:**
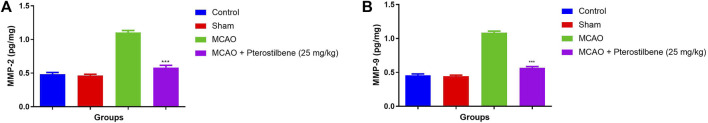
showed the MMP level of among experimental groups. **(A)** MMP-2 and **(B)** MMP-9. Values are expressed as mean ± SEM. The *t*-test was used for the significant difference between groups: *p < 0.05, **p < 0.01, ***p < 0.005. NS, non-significant.

MCAO group showed the increased level of MMP-2 (1.13 ± 0.37 pg/mg) (Figure 12A) and MMP-9 (1.08 ± 0.89 pg/mg) (Figure 12B) as compared to different group. MCAO group treated with the pterostilbene significantly (*p* < 0.001) suppressed the level of MMP-2 (0.28 ± 0.08 pg/mg) and MMP-9 (0.56 ± 0.04 pg/mg).

### Effect of Pterostilbene on Histopathology

Table 1 demonstrated the effect of pterostilbene on the brain tissue histopathology. MCAO group rats demonstrated the development of cellular swelling, cellular disintegration, macrophage infiltration, monocyte infiltration and polymorphonuclear leucocyte degranulation. Pterostilbene treatment suppressed the cellular swelling, cellular disintegration, macrophage infiltration, monocyte infiltration and polymorphonuclear leucocyte degranulation.

**TABLE 1:** showed the histopathological index of brain.

S. NO	Pathology type	Groups			
		Normal	Sham	MCAO	MCAO + pterostilbene
1	Cellular swelling	-	+1	+3	+1
2	Cellular disintegration	-	-	+3	+2
3	Macrophage infiltration	-	+1	+3	+1
4	Monocyte infiltration	-	+1	+3	+1
5	Polymorphonuclear leucocyte degranulation	-	+1	+3	+1

## Discussion

Stroke and its related disorder cause the disability and death whole over the world. The loss of cerebral blood flow during an ischemic stroke causes metabolic, biochemical and hemodynamic alteration in the damaged brain area to shut down, resulting in brain injury and reduced or lost brain function (Wang et al., 2020b; Yuan et al., 2021b). The neuronal necrosis is aggravated by reperfusion after an ischemic insult. Although the cause of cerebral ischemic stroke is complicated, some pathogenic mechanisms have shown that increasing oxidative stress and reducing endogenous antioxidant enzymes, can restore the most harmful effects of the disease (Bu et al., 2019; Shi et al., 2021). Some studies showed that oxidate stress and inflammatory reactions play a significant role to induces the serve toxicity in the biological macromolecules, which further leading the injury in the cell and tissue (Wang et al., 2020b; Shi et al., 2021). MCAO is a well-established animal model for ischemic stroke-related brain injury in order to study about the stroke phenomena and to assess the efficacy of possible neuroprotection therapies. Furthermore, neutralizing the oxidative stress, inflammatory reaction, enhanced antioxidant intake would be a beneficial therapy for the treatment of ischemic disease (Pan et al., 2018). Herb and its phytoconstituent having long history to treat the oxidative stress and inflammatory disease. Previous studies showed that the plant and its phytoconstituent having the potent anti-inflammatory, antitumor, antidiabetic and antioxidant activity (Wang et al., 2020b; Tian et al., 2020; Shi et al., 2021). The neuroprotective effect of pterostilbene against MCAO-induced cerebral ischemia reperfusion was studied in this experimental study.

During the cerebral ischemia, start the production of oxygen free radicals (OFR) *via* enzymatic and non-enzymatic systems and later on they can attack on the polyunsaturated fatty acid (PUFA), which are commonly found in the brain cells and vascular endothelial cells (Bu et al., 2019; Yuan et al., 2021b; Shi et al., 2021). Free radical starts the lipid peroxidation reaction which further start the production of various products such as hydroxy, aldehyde (MDA), keto, inner peroxy or carbonyl radicals. Some reported suggest that oxidant and free radicals play a crucial role in the expansion of edema and disruption of BBB after the cerebral ischemia (Chen et al., 2013; Bu et al., 2019). Drugs that exhibited the neuroprotective effect *via* targeting the production of free radicals to treat the disease. MDA (an indicator of lipid peroxidation, LPO) used to estimation of LPO degree and indirectly shows the cell injury in the brain (Stegner et al., 2019; Wang et al., 2020b). According to the results, pterostilbene showed the neuroprotective effect *via* decreasing the brain lesions and edema may be related to its effect in restriction the free radical production and improved the antioxidant effect. GSH neutralize the hydroperoxides and other toxic free radicals in the brain tissue (Wang et al., 2020b; Yuan et al., 2021b). GSH is endogenous antioxidant enzymes against the oxidative stress. SOD averts formation of hydroxyl radical through catalyzing the super radicals into the H2O2. CAT detoxifies the H2O2 into the water and molecular oxygen. In the brain tissue, CAT is the 2nd line antioxidant that protect brain from oxidative injury. During the cerebral ischemia and oxidation of free radical, the activity of SOD reduced in the brain tissue and serum (Si et al., 2009; Zhang et al., 2019). Large amount of SOD in consumed for neutralize or scavenge the free radicals. Due to continues uses of the SOD to neutralize or scavenge the free radicals, the activity of SOD reduces. However, the activity of SOD indirectly reflects the level of scavenged free radicals (Si et al., 2009; Li et al., 2020). Our result clearly showed that pterostilbene may contribute to suppressing the excessive production of free radical and enhance the SOD activity in the serum and brain. Pterostilbene treated group exhibited the reduction in the MDA content and enhancement of SOD content, but the result not significant (He et al., 2019; Chen et al., 2020). On the basis of result, we can say that Pterostilbene altered the cerebral ischemia *via* scavenging pathway of LPO, not antioxidant effects.

Stroke is a primary cause of acquired impairment in adults and one of the top causes of death worldwide, with a complicated pathological process (He et al., 2019; Chen et al., 2020). Recently, the researcher targeting the neurovascular unit for the treatment of stroke. Neurovascular unit is made up to several neurons, glial cells (microglia and astrocyte), extracellular matrix (ECM), endothelial cells (vascular cells) and BBB (Pan et al., 2018). The BBB is the most important component of the neurovascular unit, and MMPs have long been thought to play a role in the breakdown of the BBB following a stroke. During the early stage of stroke, MMPs induce the various neruo vascular dysfunction includes leakage of BBB and neuronal death. Previous report showed that MMP-9 is unusually expressed in brains suffering from cerebral ischemia injury and its enhance the brain injury and breakdown of BBB (Li et al., 2017). The patients suffer from the ischemic stroke exhibit the enhance level of MMP (MMP-2 and MMP-9) in the circulation (Gu et al., 2013; Li et al., 2017). The increase level of MMP-9 in the circulation related with the poorer prognosis. The reduction of MMP-9 in the circulation and gene considerably decreased the bleeding complications and infarct size (Li et al., 2017). Our result showed that pterostilbene may protect the BBB permeability *via* suppressing of MMP-9. Studies that used MMP inhibitors before a stroke, showed similar benefits, which matched our findings.

The recruitment of systemic macrophages and endogenous microglia indicates an early inflammatory response in cerebral ischemia injury. Inflammatory cells interact with the cerebral stimulus through production of pleiotropic mediators such as prostanoids, cytokines and chemokines (Chen et al., 2020; Li et al., 2020). The inflammatory or immune response following a stroke includes changes in numerous pro and anti-inflammatory cytokines and chemokines in the brain tissue. Previous reports suggest that targeting the inflammatory cytokines is beneficial for the treatment of cerebral ischemia (Li et al., 2020). Inflammatory reaction initiates the tissue lesions such as accumulation of free radicals as well targeting the toxic enzyme of brain tissue. Report suggest that various inflammatory mediators interact with each other and enhance the brain injury. Increases the level of inflammatory cytokines in the brain and begins the breakdown of BBB during cerebral ischemia (Gong et al., 2014; Li et al., 2020).

Report suggests that the TNF-α is activated during the brain ischemia. TNF-α generated through monocytes and macrophages and its over generated due to activation of inflammation related cells during the cerebral ischemia, that boost the local inflammation reaction in the brain tissue (Chen et al., 2020; Li et al., 2020). The level of cytokines increased as a result of leukocyte leakage into the circulation, causing an influx of neutrophils, macrophages, and microglia to enter the ischemic area, causing neuronal cell death and injury. TNF-α induces the cerebral endothelial cells injury and also enhance the BBB permeability, thus contributing to the formation of brain edema (Gong et al., 2014; Li et al., 2020). Moreover, the antiedematous effect of pterostilbene due to reduction the synthesis of TNF-α and also provide the protection of the BBB against disruption. Other inflammatory cytokines such as IL-6 and IL-1β and NF-κB activation play an important role in the pathogenesis of brain edema (He et al., 2017; Hou et al., 2018; Li et al., 2020). Recently report suggest that pterostilbene considerably suppressed the inflammatory cytokines and activated the NF-κB in the endothelial cells and lipopolysaccharide induced lung injury (Liu et al., 2016). All these results suggest that the preventive effect of pterostilbene, at least in part, from suppression the cytokines production and subsequently their signaling pathways. More molecular research needs to elucidate these possibilities.

During cerebral ischemic injury, enhance the level of iNOS due to development of injury cause by inflammatory cytokines and mediators (Li et al., 2017; Zhang et al., 2020). During the normal process, NO generated from the oxidation of L-arginine and further catalysed through synthesis of nitric oxide. The iNOS level boosted in the cerebral ischemic group, confirm the expansion of inflammation and pterostilbene considerably suppressed the iNOS level and confirm the reduction in the inflammatory reaction. Inflammatory mediators and cytokines increased the level of immunocyte such as neutrophils and macrophages (Gong et al., 2014; Li et al., 2017; Zhang et al., 2020). The boosted level of iNOS, PGE2 and COX-2, observed after the cerebral ischemia, which further expand the brain injury *via* inducing the damage in the neuronal cells (Gong et al., 2014; Li et al., 2021).

Prostaglandin is formed when arachidonic acid (AA) is metabolised, and it has been shown to have both a pro and anti-inflammatory effect in mammalian systems, as well as other physiological activities (Liang et al., 2020; Yuan and Zhang, 2021). COX is thought to have a role in brain homeostasis, such as blood flow regulation. Due to COX’s important involvement in vasodilation, rodents lacking the enzyme were more prone to stroke. COX is divided into two isomers (COX-1 and COX-2), which have similar catalytic actions but differing physiological functions (Liang et al., 2020). COX-2 is an inducible COX that catalyses the first committed step in prostaglandin production from arachidonic acid (Liang et al., 2020; Yuan et al., 2021a). COX-2 levels and PG production increase in endothelial cells, neurons, and glial cells in the brain tissue in response to diverse stimuli such as inflammatory mediators, excitatory synaptic activity, hypoxia, and growth factors. Previous report suggest that the COX-2 enzymatic activity involved in the stroke animal model that leads to the enhanced stroke damage and neuronal death (Wei et al., 2016; Liang et al., 2020). The use of pharmacological or genetic techniques to suppress COX-2-dependent PG production reduces infarct volume. As a result, a major effort is ongoing to investigate how to minimise the PG receptor pathways that cause COX-2-mediated cerebral damage after stroke. COX-2 levels are higher in infarcted human brains, according to clinical and prior studies, and it is found in both glial and neuronal cells. Previous research suggests that the enhanced level of COX-2 infiltrating the vascular cells, neutrophils and neurons in the peri-infarct zone (Abd El-Aal et al., 2013; Yu et al., 2015; Wei et al., 2016). It is well documented that COX-2 play an important role to providing the protection of brain tissue against cerebral ischemic injury during the brain injury in the rodents. The higher level of COX-2 rodent having higher infarcts volume after experimental stroke. In animal models, selective pharmacologic suppression of COX-2 activity has shown to be a *via*ble therapeutic target for stroke. Prostanoids (prostaglandin E2 and prostaglandin D2) formed through the COX pathway. (Liang et al., 2020; Yuan et al., 2021a). The level of PGE2 have been boosted in the brain after the cerebral ischemia (Liang et al., 2020). Following doubts regarding the safety of COX-2 inhibitors in 2004, most research on the role of the cyclooxygenase system in stroke focused on the PGE2 and PGD2 receptors (Ahmad and Graham, 2010). The actions of PGE2 receptors are triggered by four G-protein coupled receptors, which are the mediators of stroke injury (Liang et al., 2020; Yuan and Zhang, 2021). The level of PGE2 rose during cerebral ischemia due to the production of brain damage. Our control group rats showed a similar finding, with pterostilbene administration significantly suppressing COX-2 and PGE2, indicating an anti-inflammatory effect (Duan et al., 2016; Sotomayor-Sobrino et al., 2019). NF-κB is another significant transcription factors which activates after the reperfusion of cerebral ischemia (Simão et al., 2012; Yuan et al., 2021a). It is well known that oxidative stress/ROS activates the NF-κB signaling pathway that are involved in the various inflammatory reaction initiate after the cerebral ischemia. NF-κB play a crucial role in implementation of various inflammatory reactions that induces the brain injury during the cerebral ischemia (Abd El-Aal et al., 2013; Wei et al., 2016). NF-κB triggered the various parameters such as inflammatory cytokines and inflammatory mediators that are involved in the brain injury during the cerebral ischemia injury (Liang et al., 2020). Targeting the NF-κB is the novel approach to treat the cerebral ischemia. In this study, pterostilbene considerably suppressed the level of COX-2, PGE2 and NF-κB, suggesting the anti-inflammatory potential against cerebral ischemia.

## Conclusion

In conclusion, the current investigation showed that pterostilbene has beneficial and protective effect against cerebral ischemia reperfusion injury. Pterostilbene significantly reduced the brain edema, infarct volume and neurological score. Pterostilbene maintain the liver enzymes, renal and lipid parameters at a baseline level. Pterostilbene considerably altered the level of antioxidant enzymes in the brain tissue and reduces the oxidative stress. Pterostilbene significantly suppressed the level of inflammatory cytokines, inflammatory mediators and increased the level of anti-inflammatory cytokines. These findings show that pterostilbene may be an effective treatment for cerebral ischemic stroke. It also emphasises pterostilbene’s clinical applications in the treatment of cerebral ischemia reperfusion.

## Data Availability

The raw data supporting the conclusions of this article will be made available by the authors, without undue reservation.

## Abbreviations

MCAO, Middle cerebral artery occlusion; GSH, Glutathione; GPx, Glutathione peroxidase; SOD, Superoxide dismutase; MDA, Malonaldehyde; 8-OHdG, 8-Hydroxy-2′-deoxyguanosine; COX-2, Cyclooxygenase-2; iNOS Inducible nitric oxidase synthase; PGE2, Prostaglandin; MMP, Metalloproteinases; NVU, Neurovascular unit; BBB, Blood brain barrier; ECM, Extracellular Matrix; WHO, World Health Organization; ROS, Reactive oxygen species; NO, Nitric oxide; H2O2, Hydrogen peroxide; O2, Superoxide anion; NF-Κb, Nuclear factor kappa B; Tcm, Traditional Chinese medicine; CRP, C-reactive protein; LDH, Lactate dehydrogenase; HDL-c, High-density lipoprotein cholesterol; TG, Triglyceride; TC, Total cholesterol; LDL-c, Low-density lipoprotein cholesterol; VLDL-c, Very low-density lipoprotein cholesterol; BUN, Blood urea nitrogen; ALP, Alkaline phosphatase; ALT, Alanine aminotransferase; AST, Aspartate aminotransferase; IL-1β, Interleukin-1β; IL-4, Interleukin-4; TNF-α, Tumor necrosis factor-α; IL-6, Interleukin-6; IL-10, Interleukin-1; IL-1, Interleukin-1; ELISA, Enzyme-Linked Immunosorbent Assay Kits; SEM, Standard error mean; PUFA, Polyunsaturated fatty acid; LPO, Lipid peroxidation.

## References

[B1] Abd El-Aal S. A. El-Sawalhi M. M. Seif-El-Nasr M. Kenawy S. A. (2013). Effect of Celecoxib and L-NAME on Global Ischemia-Reperfusion Injury in the Rat Hippocampus. Drug Chem. Toxicol. 36, 385–395. 10.3109/01480545.2012.749270 23298270

[B2] Acharya J. D. Ghaskadbi S. S. (2013). Protective Effect of Pterostilbene against Free Radical Mediated Oxidative Damage. BMC Complement. Altern. Med. 13(1), 238. 10.1186/1472-6882-13-238 24070177 PMC3849269

[B3] Ahmad M. Graham S. H. (2010). Inflammation after Stroke: Mechanisms and Therapeutic Approaches. Transl. Stroke Res. 1, 74–84. 10.1007/s12975-010-0023-7 20976117 PMC2956985

[B4] Alva-Díaz C. Huerta-Rosario A. Pacheco-Barrios K. Molina R. A. Navarro-Flores A. Aguirre-Quispe W. (2020). Neurological Diseases in Peru: a Systematic Analysis of the Global burden Disease Study. Arq. Neuropsiquiatr. 78, 282–289. 10.1590/0004-282x20200018 32490965

[B5] Bhatt P. C. Verma A. Al-Abbasi F. A. Anwar F. Kumar V. Panda B. P. (2017). Development of Surface-Engineered PLGA Nanoparticulate-Delivery System of Tet1-Conjugated Nattokinase Enzyme for Inhibition of Aβ40 Plaques in Alzheimer's Disease. Int. J. Nanomed. 12, 8749–8768. 10.2147/IJN.S144545 PMC5732557 29263666

[B6] Bhatt P. C. Pathak S. Kumar V. Panda B. P. (2018). Attenuation of Neurobehavioral and Neurochemical Abnormalities in Animal Model of Cognitive Deficits of Alzheimer's Disease by Fermented Soybean Nanonutraceutical. Inflammopharmacol 26, 105–118. 10.1007/s10787-017-0381-9 28791538

[B7] Bu J. Shi S. Wang H.-Q. Niu X.-S. Zhao Z.-F. (2019). Acacetin Protects against Cerebral Ischemia-Reperfusion Injury via the NLRP3 Signaling Pathway. Neural Regen. Res. 14, 605. 10.4103/1673-5374.247465 30632500 PMC6352603

[B8] Chen X.-m. Chen H.-s. Xu M.-j. Shen J.-g. (2013). Targeting Reactive Nitrogen Species: A Promising Therapeutic Strategy for Cerebral Ischemia-Reperfusion Injury. Acta Pharmacol. Sin. 34, 67–77. 10.1038/aps.2012.82 22842734 PMC4086503

[B9] Chen X. Yao Z. Peng X. Wu L. Wu H. Ou Y. (2020). Eupafolin Alleviates Cerebral Ischemia/reperfusion Injury in Rats via Blocking the TLR4/NF-κB Signaling Pathway. Mol. Med. Rep. 22, 5135–5144. 10.3892/mmr.2020.11637 33173992 PMC7646971

[B22] Crofts A. Kelly M. E. Gibson K. L. (2020). Imaging Functional Recovery Following Ischemic Stroke: Clinical and Preclinical fMRI Studies. J. Neuroimaging 30 (1), 5–14. 10.1111/jon.12668 31608550 PMC7003729

[B10] Duan X. Wen Z. Shen H. Shen M. Chen G. (2016). Intracerebral Hemorrhage, Oxidative Stress, and Antioxidant Therapy. Oxid. Med. Cell Longev. 2016, 1–17. 10.1155/2016/1203285 PMC4848452 27190572

[B11] Gong G. Xiang L. Yuan L. Hu L. Wu W. Cai L. (2014). Protective Effect of Glycyrrhizin, a Direct HMGB1 Inhibitor, on Focal Cerebral Ischemia/reperfusion-Induced Inflammation, Oxidative Stress, and Apoptosis in Rats. PLoS One 9, e89450. 10.1371/journal.pone.0089450 24594628 PMC3942385

[B12] Gresoiu M. Christou S. (2020). Hypoxic Ischaemic Brain Injury. Anaesth. Intensive Care Med. 21, 298–304. 10.1016/j.mpaic.2020.03.009

[B13] Gu J.-H. Ge J.-B. Li M. Xu H.-D. Wu F. Qin Z.-H. (2013). Poloxamer 188 Protects Neurons against Ischemia/Reperfusion Injury through Preserving Integrity of Cell Membranes and Blood Brain Barrier. PLoS One 8, e61641. 10.1371/journal.pone.0061641 23613890 PMC3628995

[B14] Gupta Y. K. Briyal S. Gulati A. (2010). Therapeutic Potential of Herbal Drugs in Cerebral Ischemia. Indian J. Physiol. Pharmacol. 54 (2), 99–122. 10.25259/IJPP_90_2010 21090528

[B15] Han X. R. Wen X. Wang Y. J. Wang S. Shen M. Zhang Z. F. (2018). Retracted : Protective Effects of microRNA‐431 against Cerebral Ischemia‐reperfusion Injury in Rats by Targeting the Rho/Rho‐kinase Signaling Pathway. J. Cel. Physiol. 233, 5895–5907. 10.1002/jcp.26394 29227541

[B16] He Q. Li Z. Wang Y. Hou Y. Li L. Zhao J. (2017). Resveratrol Alleviates Cerebral Ischemia/reperfusion Injury in Rats by Inhibiting NLRP3 Inflammasome Activation through Sirt1-dependent Autophagy Induction. Int. Immunopharmacol. 50, 208–215. 10.1016/j.intimp.2017.06.029 28683365

[B17] He J. Li H. Li G. Yang L. (2019). Hyperoside Protects against Cerebral Ischemia-Reperfusion Injury by Alleviating Oxidative Stress, Inflammation and Apoptosis in Rats. Biotechnol. Biotechnol. Equip. 33, 798–806. 10.1080/13102818.2019.1620633

[B18] Hou Y. Wang Y. He Q. Li L. Xie H. Zhao Y. (2018). Nrf2 Inhibits NLRP3 Inflammasome Activation through Regulating Trx1/TXNIP Complex in Cerebral Ischemia Reperfusion Injury. Behav. Brain Res. 336, 32–39. 10.1016/j.bbr.2017.06.027 28851669

[B19] Jin F. M. Zhang Z. X. Wang Y. Zhao H. R. Yang Y. Y. Huang X. (2016). Protective Effect of Ento-I Plastic against Cerebral Ischemia-Reperfusion Injury in Rats 12, 947. J. Int. Pharm. Res. 10.13220/j.cnki.jipr.2016.03.019

[B20] Kumar V. Sachan R. Rahman M. Rub R. A. Patel D. K. Sharma K. (2021). Chemopreventive Effects of *Melastoma malabathricum* L. Extract in Mammary Tumor Model via Inhibition of Oxidative Stress and Inflammatory Cytokines. Biomed. Pharmacother. 137, 111298. 10.1016/j.biopha.2021.111298 33761590

[B21] Lake E. M. R. Bazzigaluppi P. Mester J. Thomason L. A. M. Janik R. Brown M. (2017). Neurovascular Unit Remodelling in the Subacute Stage of Stroke Recovery. Neuroimage 146, 869–882. 10.1016/j.neuroimage.2016.09.016 27664828

[B23] Leung S. W. Lai J. H. Wu J. C.-C. Tsai Y.-R. Chen Y.-H. Kang S.-J. (2020). Neuroprotective Effects of Emodin against Ischemia/Reperfusion Injury through Activating Erk-1/2 Signaling Pathway. Ijms 21, 2899. 10.3390/ijms21082899 PMC7215870 32326191

[B24] Li W. Suwanwela N. C. Patumraj S. (2017). Curcumin Prevents Reperfusion Injury Following Ischemic Stroke in Rats via Inhibition of NF-κB, ICAM-1, MMP-9 and Caspase-3 Expression. Mol. Med. Rep. 16, 4710–4720. 10.3892/mmr.2017.7205 28849007 PMC5647023

[B25] Li L. Sun L. Qiu Y. Zhu W. Hu K. Mao J. (2020). Protective Effect of Stachydrine against Cerebral Ischemia-Reperfusion Injury by Reducing Inflammation and Apoptosis through P65 and JAK2/STAT3 Signaling Pathway. Front. Pharmacol. 11, 64. 10.3389/fphar.2020.00064 32132924 PMC7041339

[B26] Li F. Xu Y. Li X. Wang X. Yang Z. Li W. (2021). Triblock Copolymer Nanomicelles Loaded with Curcumin Attenuates Inflammation via Inhibiting the NF-Κb Pathway in the Rat Model of Cerebral Ischemia. Ijn 16, 3173–3183. 10.2147/IJN.S300379 34007172 PMC8121676

[B27] Liang W. Lin C. Yuan L. Chen L. Guo P. Li P. (2019). Preactivation of Notch1 in Remote Ischemic Preconditioning Reduces Cerebral Ischemia-Reperfusion Injury through Crosstalk with the NF-Κb Pathway. J. Neuroinflamm. 16. 10.1186/s12974-019-1570-9 PMC6747758 31526384

[B28] Liang S. Chen Z. Li H. Cang Z. Yin K. Wu M. (2020). Neuroprotective Effect of Umbelliferone against Cerebral ischemia/Reperfusion Induced Neurological Deficits: *In-Vivo* and In-Silico Studies. J. Biomol. Struct. Dyn. 0, 1–11. 10.1080/07391102.2020.1780153 32552356

[B29] Liu J. Fan C. Yu L. Yang Y. Jiang S. Ma Z. (2016). Pterostilbene Exerts an Anti-inflammatory Effect via Regulating Endoplasmic Reticulum Stress in Endothelial Cells. Cytokine 77, 88–97. 10.1016/j.cyto.2015.11.006 26551859

[B30] Ma R. Xie Q. Li Y. Chen Z. Ren M. Chen H. (2020). Animal Models of Cerebral Ischemia: A Review. Biomed. Pharmacother. 131, 110686. 10.1016/j.biopha.2020.110686 32937247

[B31] Mendes de Cordova C. M. de Santa Helena E. T. Galgowski C. Figueira V. H. Setter G. B. Markus M. R. P. (2018). Evaluation of a New Equation for LDL-C Estimation and Prediction of Death by Cardiovascular Related Events in a German Population-Based Study Cohort. Scand. J. Clin. Lab. Invest. 78, 187–196. 10.1080/00365513.2018.1432070 29517392

[B32] Michalski D. Hofmann S. Pitsch R. Grosche J. Härtig W. (2017). Neurovascular Specifications in the Alzheimer-like Brain of Mice Affected by Focal Cerebral Ischemia: Implications for Future Therapies. Jad 59, 655–674. 10.3233/JAD-170185 28671120

[B33] Özön A. Ö. Cüce F. (2020). Clinical and Radiological Evaluation of Epilepsy after Ischemic Cerebrovascular Disease. Gulhane Med. J., 62(2):126–130. 10.4274/gulhane.galenos.2019.998

[B34] Pan Z. Cui M. Dai G. Yuan T. Li Y. Ji T. (2018). Protective Effect of Anthocyanin on Neurovascular Unit in Cerebral Ischemia/Reperfusion Injury in Rats. Front. Neurosci. 12. 10.3389/fnins.2018.00947 PMC6297832 30618576

[B35] Pandey P. Bhatt P. C. Rahman M. Patel D. K. Anwar F. Al-Abbasi F. (2018). Preclinical Renal Chemo-Protective Potential of Prunus Amygdalus Batsch Seed Coat via Alteration of Multiple Molecular Pathways. Arch. Physiol. Biochem. 124, 88–96. 10.1080/13813455.2017.1364773 28835129

[B36] Patel P. Barve K. Bhatt L. K. (2020). Narirutin-rich Fraction from Grape Fruit Peel Protects against Transient Cerebral Ischemia Reperfusion Injury in Rats. Nutr. Neurosci. 2020, 1–10. 10.1080/1028415X.2020.1821518 32965176

[B37] Peng T. Jiang Y. Farhan M. Lazarovici P. Chen L. Zheng W. (2019). Anti-Inflammatory Effects of Traditional Chinese Medicines on Preclinical *In Vivo* Models of Brain Ischemia-Reperfusion-Injury: Prospects for Neuroprotective Drug Discovery and Therapy. Front. Pharmacol. 10, 204. 10.3389/fphar.2019.00204 30930774 PMC6423897

[B38] Shamim M. Khan N. I. (2019). Neuroprotective Effect ofPanax Ginsengextract against Cerebral Ischemia-Reperfusion-Injury-Induced Oxidative Stress in Middle Cerebral Artery Occlusion Models. Facets 4, 52–68. 10.1139/facets-2018-0025

[B39] Shi L. Liu Q. Tang J.-h. Wen J.-j. Li C. (2019). Protective Effects of Pterostilbene on Ulcerative Colitis in Rats via Suppressing NF-Κb Pathway and Activating PPAR-γ. Eur. J. Inflamm. 17, 205873921984015. 10.1177/2058739219840152

[B40] Shi C. X. Ding Y. B. Yu F. Jin J. Li T. Ma J. H. (2021). Effects of Sevoflurane post-conditioning in Cerebral Ischemia-Reperfusion Injury via TLR4/NF-Κb Pathway in Rats. Eur. Rev. Med. Pharmacol. Sci. 22(6):1770–1775. 10.26355/eurrev_201803_14595 29630125

[B41] Si Y. l. Zhang J. y. Yan G. T. (2009). Protective Effect of Leptin against Cerebral Ischemia/reperfusion Injury in Mice. Nan Fang Yi Ke Da Xue Xue Bao 29 (4), 598–601. 10.27382/IJPP_1-_2009 19403373

[B42] Simão F. Matté A. Pagnussat A. S. Netto C. A. Salbego C. G. (2012). Resveratrol Preconditioning Modulates Inflammatory Response in the Rat hippocampus Following Global Cerebral Ischemia. Neurochem. Int. 61, 659–665. 10.1016/j.neuint.2012.06.009 22709670

[B43] Singh S. Shafi S. Shrivastav A. Khan S. Ahmad T. Khan N. A. (2020). A Review on Herbal Plants Used in Brain Ischemic and Reperfusion Injury. J. Drug Deliv. Ther. 10, 376–379. 10.22270/jddt.v10i5.4415

[B44] Sotomayor-Sobrino M. A. Ochoa-Aguilar A. Méndez-Cuesta L. A. Gómez-Acevedo C. (2019). Interacciones Neuroinmunológicas en el ictus. Neurología 34, 326–335. 10.1016/j.nrl.2016.08.003 27776957

[B45] Stegner D. Klaus V. Nieswandt B. (2019). Platelets as Modulators of Cerebral Ischemia/Reperfusion Injury. Front. Immunol. 10, 2505. 10.3389/fimmu.2019.02505 31736950 PMC6838001

[B46] Tang X.-J. Yang M.-H. Cao G. Lu J.-T. Luo J. Dai L.-J. (2016). Protective Effect of microRNA-138 against Cerebral Ischemia/reperfusion Injury in Rats. Exp. Ther. Med. 11, 1045–1050. 10.3892/etm.2016.3021 26998035 PMC4774489

[B47] Tian F. Liu R. Fan C. Sun Y. Huang X. Nie Z. (2020). Effects of Thymoquinone on Small-Molecule Metabolites in a Rat Model of Cerebral Ischemia Reperfusion Injury Assessed Using Maldi-Msi. Metabolites 10, 27. 10.3390/metabo10010027 PMC7023359 31936061

[B48] Traystman R. J. (2003). Animal Models of Focal and Global Cerebral Ischemia. ILAR J. 44, 85–95. 10.1093/ilar.44.2.85 12652003

[B49] Tuo Q. z. Zhang S. T. Lei P. (2021). Mechanisms of Neuronal Cell Death in Ischemic Stroke and Their Therapeutic Implications. Med. Res. Rev. 10.1002/med.21817 33957000

[B50] Turner R. J. Sharp F. R. (2016). Implications of MMP9 for Blood Brain Barrier Disruption and Hemorrhagic Transformation Following Ischemic Stroke. Front. Cel. Neurosci. 10, 56. 10.3389/fncel.2016.00056 PMC4777722 26973468

[B51] Vakili A. Einali M. R. Bandegi A. R. (2014). Protective Effect of Crocin against Cerebral Ischemia in a Dose-dependent Manner in a Rat Model of Ischemic Stroke. J. Stroke Cerebrovasc. Dis. 23, 106–113. 10.1016/j.jstrokecerebrovasdis.2012.10.008 23182363

[B52] Wang Y. Ren Q. Zhang X. Lu H. Chen J. (2018). Neuroprotective Mechanisms of Calycosin against Focal Cerebral Ischemia and Reperfusion Injury in Rats. Cell. Physiol. Biochem. 45, 537–546. 10.1159/000487031 29402799

[B53] Wang K. Ru J. Zhang H. Chen J. Lin X. Lin Z. (2020a). Melatonin Enhances the Therapeutic Effect of Plasma Exosomes against Cerebral Ischemia-Induced Pyroptosis through the TLR4/NF-Κb Pathway. Front. Neurosci. 14, 848. 10.3389/fnins.2020.00848 33013286 PMC7461850

[B54] Wang Y. Xiao G. He S. Liu X. Zhu L. Yang X. (2020b). Protection against Acute Cerebral Ischemia/reperfusion Injury by QiShenYiQi via Neuroinflammatory Network Mobilization. Biomed. Pharmacother. 125, 109945. 10.1016/j.biopha.2020.109945 32028240

[B55] Wei J. Sun C. L. Liu C. Zhang Q. M. (2016). Cerebrovascular Protective Effect of Combination of Tetradrine and Atorvastatin against Cerebral Ischemia-Reperfusion Injury in Rats via Inhibition of Inflammatory Mediators. Int. J. Clin. Exp. Med. 9 (11), 22807–22813.

[B56] Wu M. Lu S. Zhong J. Huang K. Zhang S. (2017). Protective Effects of Pterostilbene against Myocardial Ischemia/Reperfusion Injury in Rats. Inflammation 40, 578–588. 10.1007/s10753-016-0504-2 28054238

[B57] Yan M. Li M. Gu S. Sun Z. Ma T. Ma X. (2020). Ginkgo Biloba Extract Protects Diabetic Rats against Cerebral Ischemia-Reperfusion Injury by Suppressing Oxidative Stress and Upregulating the Expression of Glutamate Transporter 1. Mol. Med. Rep. 21, 1809–1818. 10.3892/mmr.2020.10990 32319622 PMC7057817

[B58] Yang Y. Fan C. Wang B. Ma Z. Wang D. Gong B. (2017). Pterostilbene Attenuates High Glucose-Induced Oxidative Injury in Hippocampal Neuronal Cells by Activating Nuclear Factor Erythroid 2-related Factor 2. Biochim. Biophys. Acta (Bba) - Mol. Basis Dis. 1863, 827–837. 10.1016/j.bbadis.2017.01.005 28089584

[B59] Yang T. Feng C. Wang D. Qu Y. Yang Y. Wang Y. (2020). Neuroprotective and Anti-inflammatory Effect of Tangeretin against Cerebral Ischemia-Reperfusion Injury in Rats. Inflammation 43, 2332–2343. 10.1007/s10753-020-01303-z 32734386

[B60] Yu H. Wu M. Zhao P. Huang Y. Wang W. Yin W. (2015). Neuroprotective Effects of Viral Overexpression of microRNA-22 in Rat and Cell Models of Cerebral Ischemia-Reperfusion Injury. J. Cel. Biochem. 116, 233–241. 10.1002/jcb.24960 25186498

[B61] Yu L. Su X. Li S. Zhao F. Mu D. Qu Y. (2020). Microglia and Their Promising Role in Ischemic Brain Injuries: An Update. Front. Cel. Neurosci. 14, 211. 10.3389/fncel.2020.00211 PMC7365911 32754016

[B62] Yuan S. Zhang T. (2021). Boeravinone B Protects Brain against Cerebral Ichemia Reperfusion Injury in Rats: Possible Role of Anti-inflammatory and Antioxidant. J. Oleo Sci. 70, 927–936. 10.5650/jos.ess21037 34193669

[B63] Yuan Y. Men W. Shan X. Zhai H. Qiao X. Geng L. (2020). Baicalein Exerts Neuroprotective Effect against Ischaemic/Reperfusion Injury via Alteration of NF-kB and LOX and AMPK/Nrf2 Pathway. Inflammopharmacology 28, 1327–1341. 10.1007/s10787-020-00714-6 32418004

[B64] Yuan H. Yang Q. Yang B. Xu H. Nasif O. Muruganantham S. (2021a). Phyllanthin Averts Oxidative Stress and Neuroinflammation in Cerebral Ischemic-Reperfusion Injury through Modulation of the NF-Κb and AMPK/Nrf2 Pathways. J. Environ. Pathol. Toxicol. Oncol. 40, 85–97. 10.1615/JEnvironPatholToxicolOncol.2020036307 33639076

[B65] Yuan Q. Yuan Y. Zheng Y. Sheng R. Liu L. Xie F. (2021b). Anti-cerebral Ischemia Reperfusion Injury of Polysaccharides: A Review of the Mechanisms. Biomed. Pharmacother. 137, 111303. 10.1016/j.biopha.2021.111303 33517189

[B66] Zhang W. Song J. K. Zhang X. Zhou Q. M. He G. R. Xu X. N. (2018). Salvianolic Acid A Attenuates Ischemia Reperfusion Induced Rat Brain Damage by Protecting the Blood Brain Barrier through MMP-9 Inhibition and Anti-inflammation. Chin. J. Nat. Med. 16(3):184–193. 10.1016/S1875-5364(18)30046-3 29576054

[B67] Zhang W. F. Jin Y. C. Li X. M. Yang Z. Wang D. Cui J. J. (2019). Protective Effects of Leptin against Cerebral Ischemia/reperfusion Injury. Exp. Ther. Med. 17, 3282–3290. (Review). 10.3892/etm.2019.7377 30988703 PMC6447799

[B68] Zhang C. Chen S. Zhang Z. Xu H. Zhang W. Xu D. (2020). Asiaticoside Alleviates Cerebral Ischemia-Reperfusion Injury via NOD_2_/Mitogen-Activated Protein Kinase (MAPK)/Nuclear Factor Kappa B (NF-κB) Signaling Pathway. Med. Sci. Monit. 26, e920325. 10.12659/MSM.920325 32006420 PMC7009775

[B69] Zhang Y. Han Z. Jiang A. Wu D. Li S. Liu Z. (2021). Protective Effects of Pterostilbene on Lipopolysaccharide-Induced Acute Lung Injury in Mice by Inhibiting NF-Κb and Activating Nrf2/HO-1 Signaling Pathways. Front. Pharmacol. 11, 591836. 10.3389/fphar.2020.591836 33633565 PMC7901969

[B70] Zhao M. Hou S. Feng L. Shen P. Nan D. Zhang Y. (2020). Vinpocetine Protects against Cerebral Ischemia-Reperfusion Injury by Targeting Astrocytic Connexin43 via the PI3K/AKT Signaling Pathway. Front. Neurosci. 14, 223. 10.3389/fnins.2020.00223 32300287 PMC7142276

