# Supplementary figures and images for: Neuroprotective and Anti-Inflammatory Effect of Pterostilbene Against Cerebral Ischemia/Reperfusion Injury via Suppression of COX-2
Source: Front Pharmacol. 2021 Nov 2;12:770329. doi: 10.3389/fphar.2021.770329 (PMC8593399; doi:10.3389/fphar.2021.770329)

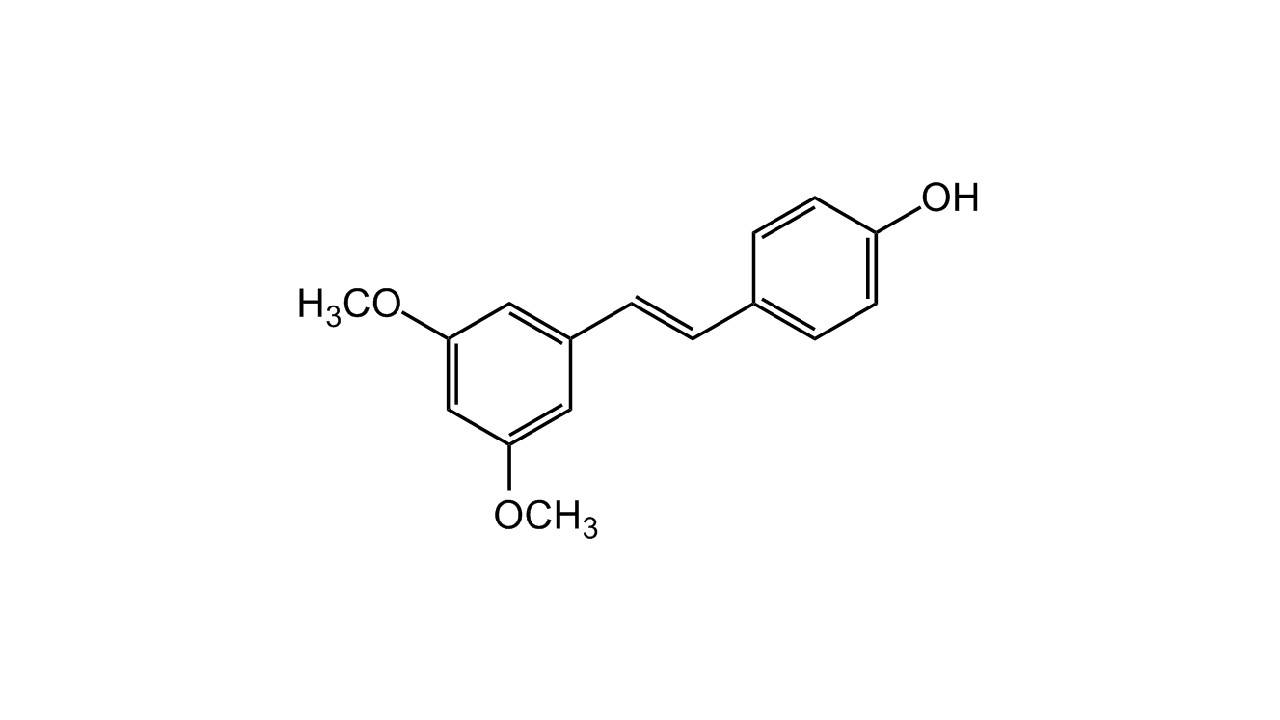

Supplement: Supplementary file 1 [file Image1.JPEG]
